# Supplementary material for: Interactive, computer-based, and situated design for innovative formative assessment approaches
Source: PLoS One. 2025 May 30;20(5):e0323301. doi: 10.1371/journal.pone.0323301 (PMC12124497; doi:10.1371/journal.pone.0323301)
Supplement: Attachment — Submitted filename: Response to Reviewers.pdf [file pone.0323301.s001.pdf]

Answer to Reviewers' Comments

Ref: **Submission ID PONE-D-24-53150**

Dear Editor,

We would like to express our gratitude to you and the reviewer for your thorough examination of the manuscript and valuable feedback. We found the comments to be helpful, and we have carefully addressed your suggestions to improve and clarify the manuscript.

During the revision process, we thoughtfully considered all recommendations. Please find below a detailed, point-by-point response, highlighted in blue.

Yours Sincerely,
Federica Morleo, Pasquale Anselmi & Alessandra Vitanza

EDITOR'S REVIEW

[1]

The introduction section mentions (line 21) "teacher collaboration and teamwork". With whom are teachers collaborating? With other teachers? Make it explicit in the introduction.

Thank you for the comment. We agree that further explanation was necessary, thus we have incorporated your suggestion by revising some sentences in the introduction and explaining information. Lines 20-23 now state the following: *'The idea of teacher collaboration and teamwork with colleagues school headteachers, students, families and external educational stakeholders is related to school improvement and effectiveness; indeed, they are considered essential pillars of teacher professionalism.'*

[2]

Line 26 "these skills are especially relevant". That paragraph provides no description of what skills are considered. It is very non-specific. Maybe provide some specific examples? Especially for primary school environments. There are some much later, in Table 1, but even those are very abstract.

Thank you for the feedback. We acknowledge the importance of emphasizing the skills selected for the study. 'These skills' refers to the skills underlying collaboration. Thus, to clarify, we added the following paragraph (lines 26-30): *'Skills such as perspective-taking, conflict resolution, negotiation, and the ability to promote inclusive environments are just a few examples within the framework of collaboration and cooperation. These skills are especially relevant for teachers, as they can influence the development of students' collaborative skills [8], another important goal for the future of education.'*

Furthermore, we revised Table 1 in order to provide readers with an overview of the skills considered in the CoTeSt. The updated Table 1 is provided below for the sake of convenience.

Table 1. Collaborative and teamwork skills in teacher professionalism selected for the CoTeSt.

<table>
<tr><th>Domains</th><th>Subdomains</th><th>Skills</th><th>Applications</th></tr>
<tr><td rowspan="4">Relationship</td><td rowspan="2">Effective Communication</td><td>Using proactive communication strategies</td><td>Proposing weekly task updates
Using verbal and non-verbal communication with students</td></tr>
<tr><td>Using active listening techniques</td><td>Paraphrasing a colleague’s idea
Writing key concepts during a brainstorming session</td></tr>
<tr><td rowspan="2">Conflict Resolution</td><td>Recognizing type and relevance of a conflict situations</td><td>Identifying role confusion in group tasks
Managing peer conflicts among teachers during group activities</td></tr>
<tr><td>Identifying conflict resolution strategies</td><td>Suggesting and encouraging collaborative problem-solving among students
Managing and balancing the workload among colleagues</td></tr>
<tr><td rowspan="2">Work Management</td><td>Action Planning</td><td>Identifying and planning action strategies</td><td>Developing group task timelines
Identifying a shared objective that a group of students are required to achieve
Assigning roles based on the skills possessed by individuals
Focusing on completing a project or task</td></tr>
<tr><td>Performance Monitoring and Feedback</td><td>Monitoring performance and providing feedback</td><td>Identifying unclear instructions given to students
Highlighting students’ strengths and weakness
Suggesting targeted pedagogical strategies to colleagues
Recognizing obstacles or errors in a colleague’s classroom performance</td></tr>
</table>

Accordingly, we modified lines 123-135 as follows: ‘The CoTeSt focuses on two subdomains in the relationship domain: ‘effective communication’ and ‘conflict resolution’. In the work management domain, attention is given to ‘action planning’ and ‘performance monitoring and feedback’. Furthermore, the choice of the skills to be assessed is driven by the need to focus

on those that can be observed in the individual's behaviors alone, although they involve interaction with others. Exploring collaboration and teamwork skills for teachers is challenging because teaching is a multifaceted profession. However, TALIS provides a broad perspective on teacher teamwork activities and outlines what teachers need to promote collaboration in their practice. Indeed, teachers have many opportunities to interact and collaborate with others, both formally and informally, at different levels of interdependence and coordination [8]. The six collaborative and teamwork skills selected for the CoTeSt are provided in Table 1, alongside a description of their application in teacher professionalism.'

[3]
Section "Situated Action", lines 218-248.
This section essentially describes a test in a simulated environment, which is also a scenario-based assessment. Consider using those terms ("simulated environment" and "scenario-based assessment"), as some readers might be more familiar with such terms.
Thank you for the valuable comment. We appreciate your suggestion to use terms that might be more familiar to readers. Indeed, in the revised manuscript, we have updated the title of the Section 'Situated Action' to 'Scenario-based Assessment' (line 175).
Additionally, we revised the first sentence of the section (line 175) to include the following clarification: *'The entire test is drawn on a scenario-based assessment, theoretically grounded in Situated Action Theory [52, 53].'*
We also updated the term in line 200 accordingly, which now states: *'The choice of a scenario-based assessment also arises from the need to create an engaging and motivating testing environment that immerses teachers in the narrative framework.'*
We opted for the term 'scenario-based assessment' rather than introducing 'simulated environment' because the former better captures the test design, which is inspired by real job situations. Conversely, the term 'simulated environment' might narrow the focus to an immersive and interactive environment, potentially leading to misinterpretation. Furthermore, we consistently used only the term 'scenario-based assessment' throughout the manuscript to ensure clarity and lexical consistency, avoiding potential confusion for readers. Lastly, we retained the term 'Situated' in the title of the article to emphasize the theoretical foundations of the assessment tool.

[4]
Lines 262-266
"The conversational agents respond in a pre-defined way, independent of the teacher's responses. This is supported by OECD [36] findings, which highlight that there are no significant differences in how individuals perform in a computer-based task when interacting

with computer agents compared to human interactions, especially when agent communication occurs through on screen messages."

This is very strange. On a naive interpretation, it means there is absolutely no relation between what the agent says and what the teacher says/does. For example, the agent always gives a canned message, like "you won." That does not make sense to interlocutors. That would not be a conversation. Can you provide a better description in what sense the agent/examinee 'responses' are 'independent'?

Thank you for raising this important point. We noticed that our paragraph may have been unclear.

The *item* illustrates an interaction among colleagues, where each of them provides questions, opinions, suggestions to the others. The respondent contributes to the interaction by selecting one of the alternatives associated with the item (in multiple-choice items) or writing their responses (in short answer items). Regardless of the choice selected by the respondent, the interaction among colleagues illustrated in the following items remains coherent. Moreover, subsequent items (that is subsequent interactions among colleagues) do not depend on the choice selected by the respondent.

In that sense, the sentences of the conversational agents (Lia, Mirta, Armando) are pre-programmed but remain contextually aligned and coherent with any of the choices made by teachers. This ensures a logical narrative flow, avoiding any perception of randomness or disconnection throughout the conversation. In other words, regardless of which option teachers select, the behavior of the conversational agents remain consistent, seamlessly supporting the progression of the narrative.

The test provided the respondents with no feedback related to the fact that their responses were correct or incorrect.

For instance, an item may require teachers to choose one of three alternative themes for creating a didactic experience in their classes. The colleagues (Lia, Mirta and Armando) express their preferences, providing reasons. Then, the item asks teachers to select a response among the following options:

- *All right, Lia. Let's go with your proposal.*
- *I agree with Armando because we already have materials to work with.*
- *Beyond your personal experiences, I will choose the proposal that I find most interesting.*
- *We could decide to invent stories set in the city instead.*

Regardless of the teacher's choice, the next item begins with the following message:

> *Mirta: Great. Now we need to decide what to do with our children for the welcoming activities. The official document specifies…*

The example illustrates how the conversational agents' pre-programmed responses are carefully designed to guide participants through the narrative without disrupting the flow of the conversation.

It is worth noting that the conversational agents support multiple paths based on the diverse responses from teachers while ensuring that all participants are presented with the same set of items.

In light of this explanation, we revised lines 216-218 as follows: '*The responses of conversational agents are pre-defined, contextually aligned and coherent with any of the alternative choices selected by teachers. This ensures that all participants are presented with the same set of items.'*

Also, the OECD report meant that examinee overall performance (as indicated by scores) is similar in the different settings. It does not mean that the agent and an examinee can "talk past each other", or does it?

Regarding the OECD findings, we have carefully revised the sentence to clarify potential ambiguity in the original paragraph, as follows (lines 219-224): '*With regard to this aspect, the OECD [36] findings indicate that examinees' performance, as measured by task scores, is comparable between computer-based tasks and human interactions. This suggests that well-structured, pre-defined interactions mediated by agents (e.g., through on-screen messages) can effectively simulate the conditions of human interaction within the assessment, ensuring coherence and contextual relevance.*'

In other words, no behavioral difference emerges, regardless of whether the agents behind the screen are human or not.

[5]

Line 306, "short-free formats". This is not a common term in English. The common terms are "free response", "constructed response" and "short answer".

Thank you. In response to this concern, we have replaced the term 'short-free formats' with 'short answer' in line 329. Similarly, we also changed the term 'short-response format' to 'short answer' in the abstract.

[6]

A major problem with current manuscript is as follows.

The manuscript describes a test, but the test is situated in a simulated environment, and that simulated environment is barely described. The situations and the tasks are not described. Are those classroom tasks with (simulated) children or more administrative tasks, like “participation in a national call for proposal”. Those are very different tasks, different skills, and of different importance to educators. So the contents of the simulated scenario are crucial for understanding the novel test.

But very little is provided. Line 304 mentions “engaging them in typical role-play scenarios.” Where is a detailed description of such scenarios? In what sense are they ‘typical’?

There are some very brief glimpses, like on lines 309-314, and the rest of the section, up to line 387, and Fig.3.

Thank you for the comment. We recognize the importance of providing a detailed description of the simulated environment and the tasks embedded in the test. To address your suggestion, we made two modifications.

First, we modified the title of the section from ‘Assessment Structure’ to ‘Assessment Scenario’ (line 339).

Second, to enhance clarity and avoid potential confusion, we revised some lines and Table 2 to provide a more detailed description of the simulated environment. The updated section now states (lines 349-363): ‘*Therefore, Table 2 presents a schematic overview of the assessment scenario, detailing the situational context in which the assessed skills are applied. As shown in the table, the assessment scenario consists of five core dimensions: environment, group composition, role of members, medium format, and task content. The scenario is set in a school environment, reflecting the context of formal teaching. Teachers are required to demonstrate their skills in various interactional settings, as indicated by group composition: individually, in pairs, in groups of four, and in a classroom setting. Within these interactions, the roles of members are designed to be both symmetrical (i.e., interacting with colleagues) and asymmetrical (i.e., hierarchical relationships with headteachers or students). The medium format integrates technological tools, including chat-based, video-based, and email-based interactions, to emulate authentic professional communication. The task content outlines a wide range of teaching practices (selected based on TALIS findings [8]) that require teachers to apply their skills in realistic professional contexts.*’

Table 2. Assessment scenario's dimensions considered for the CoTeSt

**Dimensions**	**States**
Environment	Formal teaching in school
Group composition	Individual<br>Pairs<br>Groups of four<br>Classroom
Role of members	Symmetrical (colleagues)<br>Symmetrical (headteacher, students)
Medium format	Chat-based interactions<br>Video-based interactions<br>Email-based interactions
Task content	Planning lessons in teams<br>Engaging in cross-class activities<br>Providing feedback to both students and colleagues<br>Dealing with students<br>Participating in school-wide collegial activities<br>Sending formal emails to the headteacher<br>Discussing work with school colleagues

We hope this revision improves the clarity and completeness of the manuscript.

[7]
Another aspect that remains confusing is this: is the test administered as part of a full scenario-based simulation, or is it more a test-session that is 'lightly decorated' with scenario-like visuals and chat pieces? Seems more like the latter (according to lines 391-409). Overall, the presentation of the test situation and contents is not clear enough.
Thank you for the feedback. We confirm that the test was designed with scenario-like visuals, chat, video and email elements, rather than as a full simulated environment. Nevertheless, this choice was intentional, as the study is an experimental pilot, and implementing a fully immersive simulation would have required additional resources and time. However, given the promising results, we plan to develop a full simulated simulation in future works. While we acknowledge that our approach could currently represent a limitation, it could also be seen as a strength. Indeed, if the test effectively promoted authentic engagement by immersing teachers in the narrative framework even in this version, a more immersive situation could produce extreme results.

Accordingly, we have modified lines 607-612, as follows: '*For instance, the CoTeSt was implemented with scenario-like visuals, chat, video, and email elements. Undoubtedly, a possible next step could be to develop a customized computer-based application that simulates real-time chat interactions among agents. This would create a fully immersive, interactive and practical assessment experience, enhancing the tool's ability to assess collaboration and teamwork skills more authentically*.'

[8]
Line 459
"The absolute goodness-of-fit" Please describe in detail what measured variable was fitted to what independent variable. It may seem obvious to authors, but not to readers.
Thank you for the suggestion. We acknowledge that additional details were needed to clarify the test validation process.

A test developed within CbKST is a theoretical model of the association between items and skills. As such, it needs to be empirically validated on teachers' responses to test items. Within the framework of CbKST, a probabilistic model that can be used for this aim is the CBLIM. The absolute goodness-of-fit of the CBLIM based on a specific competence model (as illustrated in Table 1) has been assessed using Pearson's Chi-square statistic by comparing the observed responses provided by teachers to each item and the expected responses derived from the item-skill assignment. As outlined in lines 305-306, the goodness-of-fit is satisfactory if the *p*-value of the Pearson's Chi-Square statistic is > .1.

Accordingly, lines 301-306 have been refined as follows: *'These associations between items and skills, as defined in the competence model, represent a theoretical model that needs to be validated on empirical data. A probabilistic model that can be used to this end is the Competence-Based Local Independence Model (CBLIM) [69,70]. The absolute goodness-of-fit of the model to the data can be assessed using Pearson's Chi-square statistic. Goodness-of-fit is satisfactory if the p-value of the Pearson's Chi Square statistic is > .1.'*

Furthermore, lines 498-504 have been modified with the following clarification: *'To this end, the absolute goodness-of-fit of the CBLIM was assessed by comparing the observed response patterns of teachers (i.e., the responses provided by the teachers to the items, coded as correct/incorrect) with the expected response patterns derived from the item-skill assignment in Fig 1. Pearson's Chi-square statistic showed that the empirical data did not significantly deviate from the expected ones (p-value = .37), indicating that the goodness-of-fit is satisfactory.'*

Accordingly, we have also added clarification on how the items are coded in lines 328-331: *'Each item is associated with one or more skills, as depicted in Fig 1, and the responses are dichotomously scored as either correct or incorrect'*.

[9]

concerning "test validation" (section "Results of Quantitative Analysis")

It is absolutely unclear in what sense the test was 'validated'. The skills and their mapping to items were predefined. What is the validity notion in this case? In what sense Chi-square statistic is applicable here. Please describe explicitly.

Thank you for your feedback. We regret that this concept was unclear and we are pleased to provide a more detailed exploration of the validation process.

The empirical validation verifies that the items actually require the skills they are associated with to be solved. In our study, we developed 20 items, each of which was thought to require particular skills to be solved, as depicted in Fig 1. However, this does not ensure that respondents actually mobilize the hypothesized skills to solve the items (for instance, they could employ alternative solution strategies beyond the hypothesized skills). Therefore, administering the test is necessary to determine which skills are effectively used by participants when responding to the items.

With respect to the Chi-Square statistic, it compares the observed responses of teachers with their expected responses derived from the item-skill assignment. When the observed responses closely align with the expected ones, the model shows a satisfactory goodness-of-fit (p-value > .1), indicating that the item-skill assignment is empirically supported. Conversely, large differences between the observed and the expected responses suggest that the association between items and skills need further refinement.

In line with this, we have revised the manuscript accordingly by editing the following lines (489-504): *'As outlined in Section Competence-based Knowledge Space Theory, the competence model behind the assessment tool needed to be validated using empirical data. For this purpose, the data collected from the sample of 139 teachers were analyzed. Each item was thought to require particular skills to be solved, as depicted in Fig 1. However, this does not ensure that respondents actually mobilize the hypothesized skills to solve the items (for instance, they could employ alternative solution strategies beyond the hypothesized skills). Therefore, administering the test is necessary to determine which skills are effectively used by individuals when responding to the items. The validation of the competence model on empirical data provides evidence that supports the hypothesized association between items and skills. To this end, the absolute goodness-of-fit of the CBLIM was assessed by comparing the observed response patterns of teachers (i.e., the responses provided by the*

teachers to the items, coded as correct/incorrect) with the expected response patterns derived from the item-skill assignment in Fig 1. Pearson's Chi-square statistic showed that the empirical data did not significantly deviate from the expected ones (p-value = .37), indicating that the goodness-of-fit is satisfactory.'

[10]

Regarding merging of data (results) between the Zoom and Freelink groups, based on times of completions. What does that actually mean? That the results of the chi-square analysis represent the whole group? Would the results be different within each group?

Thank you for the insightful comment. We appreciate the opportunity to clarify the merging of data between the Zoom Group and the FreeLink Group. The results obtained from the validation process involved the entire sample of 139 teachers (comprising both groups). To better explain this choice, we added a specification in lines 508-511, which now states: *'The analyzed sample included participants who compiled the test via the Zoom platform and those who accessed it through the free link. We decided to merge the data from the two groups to ensure that the analysis reflected the response behavior of the participants, regardless of the platform used.'*

In this way, we hope to emphasize that Pearson's Chi-square statistic was calculated on the merged dataset.

Moreover, to ensure further clarity, we revised lines 511-524 as follows: *'This decision is further supported by the analysis of a common variable, such as completion times, which could provide insights into teachers' attentiveness and care while completing the test. Fig 5 shows the comparison of completion times between the Zoom Group and the FreeLink Group. Specifically, the Zoom Group spent an average of 23 minutes completing the test (SD = 7; Median = 22), whereas the FreeLink Group spent an average of 22 minutes (SD = 10; Median = 18). Outliers were not considered problematic, as some assessment steps (e.g., reading a supplementary optional document attached to the display) required additional time. The results show no statistically significant difference (with Type I error probability α = .01) in the time spent on the test, regardless of the mode of compilation (t = .58; df = 137; p-value = .56), suggesting comparable levels of engagement and attention between the groups. Nonetheless, the comparable completion times observed in both groups could indicate a consistent level of engagement and attention, thus justifying the aggregation of data into a single dataset.'*

[11]

Regarding ages and experience that were presented earlier. There is no analysis by experience groups. Maybe collapse them if needed.

Thank you for the suggestion and we hope we have understood this comment correctly. As you pointed out, there is no analysis by experience groups or age. Indeed, these characteristics were reported as descriptive features of the participants in the Section 'Sample', with the aim of better supporting the considerations proposed in the Section 'Discussion' (lines 625-632), which now states: '*Last but not least, the sample reveals that most participants in our study were teachers with only a few years of service. This may indicate that teachers in the early stages of their careers — probably influenced by recent advances in higher education and continuous training policies — are more inclined to engage with innovative educational practices, such as those illustrated in this study. This trend could reflect the enthusiasm of early-career teachers and/or the residual computer illiteracy of more experienced teachers. If the latter is the case, there is a need to implement targeted re-skilling initiatives for more experienced teachers.*'

Indeed, these considerations arise from the data we collected, and as such, we have attempted to quantify them to support our hypothesis.

[12]

Regarding the fact/finding that most of the interviewed teachers were not aware of what actually was measured (lines 517/530). This suggests that the test is a 'stealth test', and that raises some ethical considerations if such a test would be used in operational settings. That should be discussed.

Thank you for raising this important point. We recognize that the original phrasing may have led to some misunderstanding. Indeed, in our study, all participants were fully informed about the aims of the study and the use of data, and provided informed consent before compiling the test. With respect to the old section named 'Interview Result: Goals' (lines 556-568), the key consideration pertains to the perception of participants. Since the activity was framed as a research project, some participants perceived it as a professional development opportunity rather than merely an evaluation. Therefore, their lack of awareness does not stem from a lack of transparency but rather from how they engaged with the activity within the presented scenario.

We have also updated lines 424-425 accordingly, which now state: '*The study was presented as a research project aiming at improving the teaching profession*'.

To address these points, we first revised the title of the section (line 556) which now states ‘Perception’ to better reflect the focus of the findings.
Second, we updated lines 556-560 as follows: ‘*Teachers' responses focused on the pedagogical approaches employed by the conversational agents or on the educational implications of specific scenarios presented in the videos. Teachers’ reflection confirmed that they were completely immersed in the narrative framework, perceiving the activity as a professional development opportunity rather than merely as an evaluation’.*

[13]
The discussion section should discuss the abstract skills that are supposedly measured in the test and their applicability to real-life situations, their representativeness.
Thank you for the suggestion. We acknowledge the importance of discussing the importance of the assessed skills in Section ‘Discussion and Final Remarks’, thus we added the following sentences in lines 591-598 : ‘*The collaborative and teamwork skills selected for the CoTeSt are among the fundamental ones for fostering a culture of collaboration within schools, as well as for influencing classroom management and student engagement. The scenario-based assessment was designed to replicate real-life situations that teachers frequently encounter, such as mediating conflicts among colleagues, coordinating group activities, and selecting specific pedagogical strategies in team settings. The concrete application of these skills in teachers’ daily professional practice ensures an authentic assessment experience.*’

Also the notion of validity is unclear. In plain terms: what exactly do you think you are measuring and why are you sure your measures actually measure it.
Thank you for the valuable comment. We appreciate the opportunity to provide more details about the validation process, which we hope will address your feedback.

The item-skill assignment was validated using both quantitative and qualitative methods to ensure empirical robustness and conceptual coherence.
The CBLIM was employed as a quantitative validation method, as described in comments [8] and [9], and accordingly introduced in the revised version of the manuscript.
During the development of the test, relevant skills were selected (as depicted in Table 1), and the constructed items were hypothesized to require one or more of these skills to be solved. As a result, the association between items and skills was specified in Fig 1.
A qualitative method was used to check whether the items assess the hypothesized skills. First, three experts, primary school teachers from two different schools in Turin (Italy), were asked to read the test and discuss the clarity and vocabulary of items and answers.

Individual sessions were conducted, during which each teacher provided specific feedback to guide further refinements of the test.
The final version of the test was the result of this process and was approved by all the teachers involved.
The same experts, along with three additional teachers, were provided with the list of skills and were then asked to compile the test to identify the skill(s) that, according to their expertise, were required to correctly answer each item. Their evaluations confirmed the hypothesized item-skill assignment.

According to the explanation, we revised Section 'Test Validation', emphasizing the qualitative and quantitative analyses (divided into paragraphs) used to validate the competence model, along with the detailed explanation of both (lines 476-524). We also updated the structure of the whole Section 'Result' to better guide readers through the process of development and validation of the CoTeSt, which is now as follows:

- *Results*
  - *Test Design*
  - *Experimental Campaign*
  - *Test Validation*
  - *Participants' feedback*

Lines 476-504 now states:

*'**Test Validation***

The competence model of the CoTeSt was validated using both qualitative and quantitative methods to ensure empirical robustness and conceptual coherence.

***Qualitative Analysis** A qualitative method was used to check whether the items assess the hypothesized skills, as depicted in the item-skill assignment in Fig 1. Three experts, primary school teachers from two different schools in Turin (Italy), were asked to read the test and discuss the clarity and vocabulary of items and answers. Individual sessions were conducted, during which each teacher provided specific feedback to guide further refinements of the test. The final version of the test was the result of this process and was approved by all the teachers involved. The same experts, along with three additional teachers, were provided with the list of skills and were then asked to identify the skill(s) that, according to their expertise, were required to correctly answer each item. Their evaluations confirmed the hypothesized item-skill assignment.'*

Quantitative Analysis *As outlined in Section Competence-based Knowledge Space Theory, the competence model behind the assessment tool needed to be validated using empirical data. For this purpose, the data collected from the sample of 139 teachers were analyzed. Each item was thought to require particular skills to be solved, as depicted in Fig 1. However, this does not ensure that respondents actually mobilize the hypothesized skills to solve the items (for instance, they could employ alternative solution strategies beyond the hypothesized skills). Therefore, administering the test is necessary to determine which skills are effectively used by individuals when responding to the items. The validation of the competence model on empirical data provides evidence that supports the hypothesized association between items and skills. To this end, the absolute goodness-of-fit of the CBLIM was assessed by comparing the observed response patterns of teachers (i.e., the responses provided by the teachers to the items, coded as correct/incorrect) with the expected response patterns derived from the item-skill assignment in Fig 1. Pearson's Chi-square statistic showed that the empirical data did not significantly deviate from the expected ones (p-value = .37), indicating that the goodness-of-fit is satisfactory.'*

We have also updated lines 331-333 accordingly, which now state: '*Test validation was carried out using both qualitative and quantitative methods to check whether items assess the hypothesized skills, as detailed in Section Test Validation.*'

We have also added the following sentence in the abstract: '*Test validation involved qualitative and quantitative methods, confirming that the items actually assess the skills they were assumed to evaluate*.'

[14]
Give the structured flow of the test, what is the test reliability? This is not mentioned. You don't need to show results on it (if you don't have any yet), but do discuss this aspect.
Thank you for the feedback and the opportunity to discuss the aspects related to test reliability. In the framework of CbKST, the concept of reliability is relatively recent and still underexplored. Within the CBLIM, test reliability takes into account two probabilities estimated for each item, defined as careless error and lucky guess. The former is the probability that an individual fails the item despite mastering all the skills needed to solve it, whereas the latter is the probability that an individual solves the item despite lacking some or all the required skills. Possible reasons for these outcomes might be related to the properties of the items. For instance, the wording of the item could confuse the respondents, leading them to provide an incorrect response. In the case of multiple-choice items, respondents could guess the correct answer. Low values (i.e., < .5) for the estimated careless error and

lucky guess probabilities of an item indicate that there is a high degree of agreement between the responses an individual is expected to give, based on the skills they master, and the responses that the individual actually gives.

To address your comments, we incorporated these explanations in Section ‘Competence-based Knowledge Space Theory’, lines 304-323.
Furthermore, our findings reveal that the careless error and lucky guess probabilities of the CoTeSt items are quite low, indicating that the test is reliable. We added this specification in Section ‘Quantitative Analysis’, lines 504-507, which states: *‘Moreover, the mean of the careless error probabilities is .21, whereas that of the lucky guess probabilities is .37. These results suggest that the item-skill assignment in Fig 1 appropriately describes the skills required for solving each of the test item [28,79].’*

References

[8] Organisation for Economic Co-operation and Development (OECD). Teaching in Focus: Professional Collaboration for Teachers Working in Challenging Environments. Teaching in Focus. 2020;doi:10.1787/c699389b-en.

[28] Morleo F, Vitanza A, Anselmi P. Design of a Test for Assessing Teamwork and Collaborative Skills with Competence-Based Knowledge Space Theory. In: Proceedings of the ASA2024 Conference: Measuring and Interpreting World Changes with Statistics, Data Science and AI. Rome, Italy; 2024.

[36] Organisation for Economic Co-operation and Development (OECD). PISA 2015 Results (Volume V): Collaborative Problem Solving. Paris: OECD Publishing; 2017.

[52] Suchman LA. Plans and Situated Actions: The Problem of Human-Machine Communication. Cambridge University Press; 1987.

[53] Suchman LA. Human-Machine Reconfigurations: Plans and Situated Actions. 2nd ed. Cambridge University Press; 2007.

[69] Heller J, Stefanutti L, Anselmi P, Robusto E. On the Link Between Cognitive Diagnostic Models and Knowledge Space Theory. Psychometrika.2015;80(4):995–1019.doi:10.1007/s11336-015-9457-x.

[70] Heller J, Stefanutti L, Anselmi P, Robusto E. Erratum to: On the Link Between Cognitive Diagnostic Models and Knowledge Space Theory. Psychometrika. 2016;81(1):250–251. doi:10.1007/s11336-015-9494-5.

[79] Morleo F, Vitanza A, Anselmi P. How Can We Precisely Assess Collaborative and Teamwork Skills? Development and Validation of a Competence-based Knowledge Space Theory Test. In: Proc. of the European Mathematical Psychology Group (EMPG) Conference 2024. Tübingen, Germany; 2024.Available from: https://www.mathpsy.uni-tuebingen.de/empg2024/EMPG2024_Program&Abstracts.pdf.

AUTHOR’S REVIEW

Reviewer #1:

The manuscript presents a very interesting computer-based assessment procedure to explore different skills that are identified as essentials for pre-primary and primary teachers. Test validation is performed within the Knowledge Space Theory framework. On the one hand, I've found the paper a very interesting reading and I consider the approach followed very promising and engaging. No doubt worth of consideration for publication. On the other hand, I have found the manuscript both a bit fragmented and sometimes not detailed enough. Please let me stress that I consider the following comments more as subjective opinions rather than statements of objective issues in the work, but I still believe that they deserve some consideration on the Authors side.
Thank you for your valuable comment. We greatly appreciated your feedback and the recognition of the study’s relevance. We have carefully considered your suggestions and have addressed them in detail in the following responses.

As to the **fragmentation**, I was under the impression that splitting the content of the sections "Challenges"' and "Our proposals" was likely meant to clearly separate the literature review part from the original contribution of the Authors, but ended up splitting the reasoning behind the choices of the Authors from the actual conclusions of their reasoning. I had to jump back and forth several times between the sections because the structure was forcing me as a reader to keep in mind everything I read before concerning different aspects and topics related to the project before actually seeing the natural conclusions of the Authors' reasoning for each and every single aspect or topic. As an example, first a literature review on skills is carried out, then several other topics are discussed, only later in the new section conclusions are briefly drawn about which skills should actually be further considered based on the literature review.
Thank you for your valuable feedback which raises this important point. We acknowledge that following the logical flow of the manuscript may have been perceived as difficult according to the previous structure. Therefore, based on your insightful comment, we have reconsidered the organization of the Sections ‘Challenges’ and ‘Our Proposals’, in order to reduce fragmentation while promoting readability. The revised structure is as follows:

Challenges and Our Proposals

Challenge 1: Defining Collaborative and Teamwork Skills

Assessing Collaborative and Teamwork Skills in Teachers

Challenge 2: Teachers Assessment

Online Assessment

Simulated Environment

Conversational Agents

Challenge 3: Formative Assessment

Competence-based Knowledge Space Theory

We have also modified lines 68-70, which now explains to readers the structure of the manuscript as follows: '*Section 'Challenges and Our Proposals' examines three key challenges from a theoretical perspective and addresses each of them with the corresponding practical solution adopted in this work.*'

Accordingly, we have revised the introduction of the various sections in order to correctly link each challenge to the respective proposal.

We hope this solution could provide a more cohesive structure, allowing readers to immediately catch the reasons behind each proposal.

As to the lack of **details**, I had the impression that the Authors might have discussed more in depths the relation and differences of their work with other existing approaches to engage teachers while assessing their skills. I also had the impression that the work sometimes was a bit lacking in technical aspects as if the Authors were avoiding any technical discussion concerning the type of approach and models chosen. Maybe the Authors made this choice to try to engage the broadest possible audience, however while reading the manuscript I had several questions that I felt were basically unanswered. For instance, why KST was chosen? In KST guessing and slipping can only be performed at the item level, vs. for instance a CDM approach in which guessing and slipping might be performed at the skill level. One might wonder if in the context of evaluating teachers' skill it might be more relevant to know with how much confidence we can say that a teacher does actually possess a certain skill rather than assuming that it is there or not and any random effect falls on the items. Similarly, once the KST approach is justified and chosen it might be interesting to provide further considerations on the type of knowledge structure obtained in the assessment and on the meaning, interpretation, and actual values of guessing and slipping parameters involved in the test under the CBLIM.

Thank you for the feedback and the opportunity to discuss these aspects. While we acknowledge that additional details concerning the technical aspects of CbKST could be

beneficial, they fall beyond the scope of the present manuscript and they have been addressed in an article now cited in the manuscript:

[28] Morleo F, Vitanza A, Anselmi P. Design of a Test for Assessing Teamwork and Collaborative Skills with Competence-Based Knowledge Space Theory. In: Proceedings of the ASA2024 Conference: Measuring and Interpreting World Changes with Statistics, Data Science and AI. Rome, Italy: Association for Applied Statistics; 2024.

We would also like to confirm that this paper was written with the intention of engaging the broadest possible audience. In doing so, we aimed to provide a comprehensive overview of the challenges we encountered during the design process. The technical aspects discussed in the paper are presented as our proposals for addressing these challenges, rather than as definitive solutions. We believe that this approach allows readers from various backgrounds to appreciate the broader context while also understanding the specific technical considerations that shaped our work, especially since the evaluation of soft skills is currently an understudied area of research.

However, to address your comments, we updated the manuscript by incorporating additional technical details, while maintaining clear and accessible language. For specific aspects which were not explicitly detailed, we provided appropriate references to guide readers. In particular, we added a deeper exploration of the following aspects:

- CBLIM (lines 306-323). We introduced the meaning and interpretation of the model's results: *p*-value, careless error probability, lucky guess probability, alongside the use of specific terms (e.g., 'competence state').
- Results of the quantitative analysis of the test validation (lines 489-507). We provided the description of the results obtained in the test validation (i.e., *p*-value, careless error probability, lucky guess probability).
- Analysis of time (lines 508-524). We described the analysis of the time spent by participants of the Zoom Group and Free Link Group on the test.

Furthermore, in Section 'Competence-based Knowledge Space Theory', we described the reasons why we used CbKST in developing the CoTeSt. According to the section, CbKST was chosen because it provides a theoretical framework for assessing the skills an individual masters based on their observed responses to test items. By doing so, CbKST also addresses the growing interest in obtaining a formative assessment by describing the results derived from the evaluation.

To address your valuable considerations, we added the following sentences in lines 280-284: *'CbKST is not the only framework for assessing the skills an individual masters from the observed item responses. For instance, Cognitive Diagnostic Models [67, 68] represent a valuable alternative. It is worth mentioning that there is a close correspondence between the latter framework and CbKST, which ranges from very basic concepts to formal models [69] .'*

A few minor comments follow:

1 - lines 9-10. Could the Authors elaborate a little more on the topic and provide literature or data to back their claim up? Not that I disagree with the statement, but I think that the topic deserves a bit more of explanation and underpinning.

Thank you for the suggestion. We agree that further clarification is necessary; therefore, we have revised lines 5-10 as follows: 'Indeed, as pointed out by the OECD Reviews of School Resources [3], the qualification requirements for primary education teachers, as well as school leaders, vary considerably from country to country. Nevertheless, it is broadly acknowledged that teacher education and training should be based on autonomy and responsibility, connections with evidence-based research, and strong ties to society [2].' We hope this additional information enhances the completeness of the manuscript.

2 - lines 94-104. Given its generality and applicability to the other subsections, shouldn't the discussion on the definition of and the relation between skills and competences come before, maybe at the introduction level?

Thank you for your thoughtful suggestion. We agree that the digression about the definition of skill adopted within the manuscript should come earlier, therefore, we moved it to the introduction section, immediately after citing the relevant skills for teacher professionalism (lines 30-40).

3 -lines 159-160. I am not sure why the (Cambridge?) Center for Evaluation and Monitoring is backed up by a citation to the Sutton Trust-EEF Teaching and Learning Toolkit.

Thank you for carefully reading the manuscript. As you pointed out, there was an error in our citation. We have replaced it with the correct article published on the blog of the Cambridge Centre for Evaluation and Monitoring (CEM), titled '*A complete and effective assessment cycle*', published on November 12, 2024, available here: https://www.cem.org/blog/complete-assessment-cycle. It explores the role of formative assessment within the assessment cycle.

4 - lines 285-287. This is just a quibble, but I found the wording of this sentence slightly confusing. At first glance I thought the Authors wrote `rows' in place of `columns', then by looking at the Figure I realized that they were not giving a Q-matrix/skill map with conjunctive or disjunctive interpretations, but they were actually repeating items in different rows when

different solution strategies were present. Maybe the Authors could emphasize that the items are repeated, and maybe also add in Figure some very light separation lines between the different items so that item repetition becomes immediately visible.
Thank you for the comment. We appreciate your suggestion to better guide the reading of Fig. 1. Indeed, we have revised lines 291-294 to include the clarification: *'Some items are repeated in two different rows and are associated with two different skills. This means that there are two strategies for solving these items, each involving one of the two skills.'* Moreover, we updated Fig 1, by highlighting the presence of the same items in different rows.

5 - lines 360. Could the Authors expand on the role of fake-items and maybe provide an example on how they are needed for the narrative?
Thank you for raising this important point, which is closely related to the presence of conversational agents throughout the narrative assessment process.

In the 'simulated' chat, the sentences of the conversational agents represent the items, while the responses are the alternative choices available to teachers. The responses of the conversational agents are pre-programmed but remain contextually aligned and coherent with any of the choices made by teachers. This guarantees a logical narrative flow, avoiding any perception of disconnection throughout the conversation. By ensuring that the responses of the conversational agents remain consistent, we have designed 'fake-items', through which all participants are presented with the same set of items.
For instance, the first part of the test asks teachers to choose one of three alternative themes for creating a didactic experience in their classes. Specifically, the participant and another colleague -called Mirta- have been required to work together. Mirta sent to the participant a private message, which states:

> ***[FAKE ITEM yes/no]***
> *'What a bore. Fortunately, we have a joint hour on Tuesday with both classes. I remember your passion for the mountains... I also like it a lot. What do you think if we chose the mountain as the theme for setting up the work?*
>
> *Choose one of the proposed options as your response:*
>
> - Yes
> - No

If the participant select 'No', then the following slides will state:

> *[FAKE ITEM]*
> *Mirta: What theme would you like the story to focus on?*

Write your answer to send to Mirta….. (type your answer here)

Mirta: Great idea. It will works. (press 'Continue' to proceed with the conversation with Mirta)

[ITEM 03]
Mirta: How exhausting. all these extra hours are just more work and wasted time. (choose one of the proposed responses…)

If the participant selects 'Yes' to the 'FAKE-ITEM yes/no', the next item will be immediately item 03.
A rephrase of this example can be also seen in Fig 2 within the manuscript (lines 380-385): '*As an example, the control flow applied to three illustrative items is depicted in Fig 2. This helps clarify the functionality and necessity of fake-items. The fake-items, in fact, were introduced to guide participants through the narrative without disrupting the flow of the conversation and to allow them to explore all the alternative paths without influencing their genuine behaviors. In fact, responses to fake items were not analyzed.*'

6 - lines 433. As the Authors pointed out that the sample roughly captures the gender imbalance of the Italian educational system, I was wondering if also the prevalence of low seniority teachers in both groups is representative of the Italian system or rather to the difficulty in sampling older teachers (e.g., computer illiteracy)? Edit: I see that the Authors elaborate a bit on the topic on the final discussion but I think it would still be better if they addressed it also previously.

Thank you for your feedback and we hope to address your comment.

In the manuscript, we reported the age and work experience of participants as a characteristic of our sample, which were analyzed after data collection. Therefore, these do not necessarily reflect the broader Italian teacher population. Indeed, we have not been able to find data regarding the proportion of the age of teachers in primary school contexts. We used the descriptive features of the sample to support the considerations proposed in the Section 'Discussion and Final Remarks' (lines 625-634), which now states: ' *Last but not least, the sample reveals that most participants in our study were teachers with only a few years of service. This may indicate that teachers in the early stages of their careers — probably influenced by recent advances in higher education and continuous training policies — are more inclined to engage with innovative educational practices, such as those illustrated in this study. This trend could reflect the enthusiasm of early-career teachers and/or the residual computer illiteracy of more experienced teachers. If the latter is the case,*

there is a need to implement targeted re-skilling initiatives for more experienced teachers. Equipping all educators with the relevant skills to navigate evolving educational paradigms and social changes is essential for fostering a cohesive, adaptive, and future-ready teaching workforce."

These considerations are addressed in the Section 'Discussion and Final Remarks' because they arise from our direct experience, and as such, we have attempted to quantify them to support our hypothesis.

References

[2] Organization for Economic Co-operation and Development (OECD). TALIS 2018 Results (Volume II): Teachers and school leaders as valued professionals. OECD Publishing; 2020. Available from: https://doi.org/10.1787/19cf08df-en.

[3] OECD (2019). Working and Learning Together: Rethinking Human Resource Policies for Schools, OECD Reviews of School Resources, OECD Publishing, Paris.

[28] Morleo F, Vitanza A, Anselmi P. Design of a Test for Assessing Teamwork and Collaborative Skills with Competence-Based Knowledge Space Theory. In: Proceedings of the ASA2024 Conference: Measuring and Interpreting World Changes with Statistics, Data Science and AI. Rome, Italy: Association for Applied Statistics; 2024.

[67] Rupp AA, Templin J, Henson RA. Diagnostic Measurement: Theory, Methods, and Applications. Guilford Press; 2010.

[68] DiBello LV, Roussos LA, Stout W. Review of Cognitively Diagnostic Assessment and a Summary of Psychometric Models. In: Rao C, Sinharay S, editors. Handbook of Statistics. vol. 26. North-Holland Publications; 2007. p. 970–1030.

[69] Heller J, Stefanutti L, Anselmi P, Robusto E. On the Link Between Cognitive Diagnostic Models and Knowledge Space Theory. Psychometrika. 2015;80(4):995–1019. doi:10.1007/s11336-015-9457-x.
